# Inflammation, Cerebral Vasospasm, and Evolving Theories of Delayed Cerebral Ischemia

**DOI:** 10.1155/2013/506584

**Published:** 2013-08-22

**Authors:** Kevin R. Carr, Scott L. Zuckerman, J Mocco

**Affiliations:** ^1^Department of Neurological Surgery, University of Texas Health Sciences Center at San Antonio, San Antonio, TX 78229, USA; ^2^Department of Neurological Surgery, Vanderbilt University School of Medicine, Nashville, TN 37212, USA

## Abstract

Cerebral vasospasm (CVS) is a potentially lethal complication of aneurysmal subarachnoid hemorrhage (aSAH). Recently, the symptomatic presentation of CVS has been termed delayed cerebral ischemia (DCI), occurring as early as 3-4 days after the sentinel bleed. For the past 5-6 decades, scientific research has promulgated the theory that cerebral vasospasm plays a primary role in the pathology of DCI and subsequently delayed ischemic neurological decline (DIND). Approximately 70% of patients develop CVS after aSAH with 50% long-term morbidity rates. The exact etiology of CVS is unknown; however, a well-described theory involves an antecedent inflammatory cascade with alterations of intracellular calcium dynamics and nitric oxide fluxes, though the intricacies of this inflammatory theory are currently unknown. Consequently, there have been few advances in the clinical treatment of this patient cohort, and morbidity remains high. Identification of intermediaries in the inflammatory cascade can provide insight into newer clinical interventions in the prevention and management of cerebral vasospasm and will hopefully prevent neurological decline. In this review, we discuss current theories implicating the inflammatory cascade in the development of CVS and potential treatment targets.

## 1. Introduction

Subarachnoid hemorrhage (SAH) is a devastating neurological insult that causes significant morbidity and mortality [1]. One of the greatest sources of this morbidity and mortality is cerebral vasospasm (CVS), leading to delayed cerebral ischemia (DCI) [2]. While angiographic vasospasm is thought to occur in approximately 70% of patients after aSAH, only 25% develop symptomatic CVS [3, 4]. Morbidity remains high despite years of clinical and basic science research done on the topic, with approximately 50% infarction rates in affected patients [5, 6].

An antecedent inflammatory cascade is one of the many etiologies thought to be responsible for the development of CVS. Experimental studies have shown involvement of cytokines, cell adhesion molecules, and leukocytes, and early clinical studies have attempted to inhibit components of the inflammatory cascade to mitigate CVS [7–19]. Additionally, endothelin receptor activation, nitric oxide inhibition, thromboxane receptor modification, and many cell signaling cascades are thought to play an integral role in the development of this pathology [20–27]. Currently, the primary treatment for this patient population involves hemodynamic augmentation and medically or surgically mediated intra-arterial vasodilation. These treatments, while providing amelioration in the short term, have proved relatively ineffective in staving off neurological decline, in part due to lack of large scale analyses [1, 28]. In our review, we hope to summarize past and current research in the area of inflammation and the development of CVS. An appreciation of these key inflammatory intermediaries will allow for the development of clinically significant interventions in this patient population.

## 2. Clinical Diagnosis of CVS

In patients with aSAH, neurological dysfunction presents within several hours after the injury. This decline was initially attributed to the progression of mass effect secondary to acute SAH; however, current evidence suggests that an acute brain injury with subsequent cerebral ischemia plays a primary role. It is thought that the introduction of blood products, specifically hemoglobin, into the extravascular space initiates narrowing of the arterial vasculature within several hours [29], starting at approximately three days after-injury and lasting up to several weeks [30]. The arterial vasculature demonstrates diffuse or focal stenosis with significant restriction of blood flow visualized via angiography. CVS presents with focal neurological dysfunction and can precipitate the development of permanent ischemia in approximately 50% of patients with CVS [2].

The gold standard for the diagnosis of CVS is angiography, specifically with digital subtraction angiography (DSA) [31]. As an improvement to the technique described by Ecker and colleagues in 1951 [32], DSA allows for high quality angiographic images via the digital subtraction of extraneous artifact [33]. In its use as a diagnostic tool, mild vasospasm is defined by less than 25% reduction in vessel patency. A greater than 50% reduction is termed severe vasospasm [34]. Many authors have since espoused the utility of CTA and MRA as alternatives to the invasive techniques used in DSA [35–37]. In addition to being less invasive and severalfold faster than conventional angiography, CTA and MRA produce images of higher resolution than their predecessor [38, 39]. These techniques however rely heavily on digital reconstruction, and their accuracies vary depending on the vascular territory assessed and operator experience [31, 35, 39, 40].

Transcranial Doppler sonography (TCD) is currently the primary tool used in the noninvasive diagnosis of cerebral vasospasm. Its use was first described in 1981 when Dalessandri et al. demonstrated efficacy in the visualization of cerebral blood flow in cerebral arteries [41]. It is currently the single most important noninvasive measure of cerebral vasospasm [42, 43]. When vasospasm induces a decrease in arterial diameter, cerebral blood flow (CBF) velocity concomitantly increases. CVS is most times described when CBF velocity exceeds 120 cm/s [44, 45]. An increased ratio of CBF velocity between intracranial vessels, the middle cerebral artery (MCA) or anterior cerebral artery (ACA), and an extracranial ICA can be used to indicate CVS. This value is known as the Lindegaard index and is widely used today [46]. Most institutions identify clinical vasospasm as a ratio above 3 : 1. The diagnostic reliability of TCD has historically been called into question since factors such as the arterial window analyzed, patient movement, and operator experience affect the reliability of the test [31, 43, 47]. Specifically, changes in systemic pressure, cardiac output, and other factors that affect hemodynamic homeostasis can adversely affect reliability of the tool due to its strong dependence on cerebral blood flow dynamics [48]. In a comparative study between TCD and DSA in the assessment of aneurysmal vasospasm, Okada and colleagues highlighted the poor sensitivity of TCD for detecting vasospasm and noted that DSA was distinctly superior as a diagnostic tool [47]. Other studies have underscored the incongruences in the diagnostic sensitivity of this tool [49, 50]. Tools such as jugular venous oxygen saturation monitoring, Xe-enhanced CT, positron emission tomography (PET), single photon emission CT (SPECT), and magnetic resonance imaging have also been used to diagnose CVS with varying sensitivities [51]. 

## 3. CVS and Inflammation

### 3.1. History

The earliest correlation between CVS and inflammation was established over 60 years ago. Walton in 1955 demonstrated that febrile patients who presented with SAHs fared worse than their afebrile counterparts [52]. Several decades later, studies by Rousseaux et al. confirmed a definitive relationship between the occurrence of symptomatic CVS and fever in affected patients (Table 1) [53]. It was in the late 1980's however that Spallone and colleagues demonstrated that leukocytosis, the sentinel indicator of systemic inflammation, was significantly higher in patients with aSAH [54]. These discoveries spurred interest in the role of the inflammatory response as a causative factor in the development of the pathology. Later studies substantiated these earlier findings, and additional inflammatory markers and cytokines were identified to play a pivotal role in the inflammatory process (Table 1).

### 3.2. The Inflammatory Cascade and CVS

As a result of decades of research into the inflammatory mediators of CVS, several theories explaining the pathophysiology have been proposed. One such theory states that the extravasation of erythrocytes into the cerebral parenchyma acts as the nidus for the vasospasms that characterize acute and chronic CVS [11, 18, 55, 56]. Extracorpuscular hemoglobin (Hb) is a proinflammatory moiety as opposed to its intravascular homolog [57–60]. The presence of Hb in the extravascular space liberates reactive oxygen species (ROS) that result in the peroxidation of membrane lipids of endothelial cells and proliferation of smooth muscle [61, 62]. It is also thought that these oxidative stressors inhibit bradykinin mediated vascular relaxation after aSAH [63]. Additionally, it has been hypothesized that the presence of subarachnoid blood is a potent stimulant for nuclear factor-kappa B (NF-*κ*B) mediated cytokine release augmenting the inflammatory reaction [11, 64]. Normally, once erythrocytes have entered the extravascular space, exposed Hb is rapidly bound to a hepatically produced haptoglobin (HP). HP exists in two isoforms, HP 1-1 and HP 2-2 of which HP 2-2 is thought to bind with less affinity and as such is relatively pro-inflammatory in comparison to its analog [23, 27, 56, 65]. Macrocytes phagocytize Hb bound moieties and remove them from the extravascular space. The initial pro-inflammatory effect elicited by Hb and Hb bound moieties initiates an inflammatory cascade involving an increase of cytokines, leukocytes, and cell adhesion molecules (CAMs) characterizing the inflammatory process [7, 13, 18, 56, 66, 67].

The elevation in inflammatory cytokines and cell adhesion markers promotes the initial margination of leukocytes by CAMs on the endothelial surface. Of these cell adhesion markers, selectins (L-, P-) are most important in the initial capture [9, 20, 68–71]. Secondly, leukocyte rolling is facilitated by P- and E-selectins in which captured leukocytes are sequentially bound and released by adjacent CAMs. The end result is a sequential transmigration of leukocytes down a chemoattractant concentration gradient to the site of inflammation. Finally, firm adhesion to the endothelium effectively halts leukocyte rolling and promotes leukocyte diapedesis [11, 72]. This process is mediated by another class of CAMs, the integrins of which CAM-1, LFA-1 and other moieties play vital roles. Immunoglobulins ICAM-1, and VCAM-1 are also required for adhesion [73–75]. Experimental models have successfully demonstrated the temporal relationship between the initial inciting injury and increases in cytokine concentrations [76–78]. Of particular interest are IL-1*β*, IL-6, TL-8, and TNF-*α*. In various studies, increases in the intraventricular concentrations of these moieties have been demonstrated to occur after injury [79–81]. Though the CSF concentrations of these inflammatory intermediaries increase after-hemorrhage, the change in serum concentrations is not as reliable. In fact, studies have demonstrated both elevations [82] and no relevant changes [76] in the analysis of systemic concentration of these cytokines. It is irrefutable, however, that leukocytosis and changes in C-reactive protein levels (CRP) correlate strongly with clinical outcomes after-injury [83, 84].

Transendothelial migration, or diapedesis, is the final step in the recruitment of inflammatory leukocytes and encompasses a multitude of cell adhesion molecules and intracellular cell signaling pathways [72]. Macrophages, monocytes and neutrophils are the primary cells implicated in the initial stages of injury and have been found in postinjury histological analysis to be a highly represented cell type in adjacent tissue [85, 86]. These inflammatory cells mediate macrocytosis of free erythrocytes as part of their natural function; however, after aSAH there is a disruption of normal cerebral blood flow preventing the re-entry of macrophages and leukocytes into circulation. This results in the degradation and degranulation of these cells in the parenchymal space. Ultimately, a release of inflammatory cytokines and other vasoactive compounds, which characterize the chronic vasospasm of CVS, is thought to occur approximately four days after injury [18]. 

In the final analysis, the upregulation of ET-1, destructive free radicals, NO dysregulation, and spasmodic cytokines are all thought to play a substantial role in the onset of vasospasm [1, 87–89]. ET-1 itself is a potent vasoconstrictor whose upregulation peaks 3-4 days after injury [90]. It is thought to be increased due to cerebral ischemia and other stressors with possible augmentation by the aforementioned antecedent inflammatory cascade. Its effect on vasoconstriction is multifaceted [91, 92]. Through a negative feedback loop, its activation of endothelial nitric oxide synthetase (NOs) depletes NO concentrations [93]. NO plays a significant role in vascular tone through the inhibition of calcium production and subsequent reduction in contractile forces; thus, its inhibition leads to vessel narrowing [94]. Additionally, ET-1 is also integral to fibrosis, tissue growth, and inflammation [26, 92, 95]. In summary, through several distinct mechanisms, the inflammatory changes surrounding SAH lead to global stenosis of cerebral vasculature [26, 96–99].

### 3.3. Research Findings Relating Inflammation to CVS

Research has focused on modifying several inflammatory markers (Table 2). Several clinical trials have either corroborated or refuted the clinical efficacy of the suggested treatments [1]. Anti-inflammatory agents such as dexamethasone and cyclosporine A (Cys-A) have shown favorable improvements in experimental models [100, 101]. However, other known agents, such as tacrolimus, an inhibitor of T-cell maturation similar to Cys-A, have not shown similar results [102]. There are also studies implicating the complement system, the 3-hydroxy-3-methyl-glutaryl coenzyme A (HMG-CoA) reductase pathway, and multiple other systems; however, results even in the experimental stages have been varied [103–106].

There have been experimental reports documenting improvement in CVS using various pharmaceutical agents, however, the variety of these treatments is sparse (Table 3). In 2011, Velat and colleagues reviewed 44 randomized control trials (RCT) and 9 meta-analyses on vasospasm after SAH [1]. Their studies suggested that only oral nimodipine was efficacious in stymieing CVS with improvement in neurological decline after SAH. In an experimental murine model of cerebral vasospasm, Bowman and colleagues demonstrated a statistically significant dose dependent reduction in vasospasm after administration of a polyclonal antibody targeted against IL-6 (*P* < 0.05) [107]. Such findings indicate that cytokine upregulation is antecedent to radiographic vasospasm, and their attenuation may minimize vessel narrowing. The propensity for magnesium supplementation to mitigate CVS has also been widely studied [108–113]. In fact, a small Swiss study in 2012 suggested that high dose magnesium administration compared to low dosage suppressed post-SAH increases in IL-6 (*P* = 0.021) and IL-1*β* (*P* < 0.001), respectively [78]. 

Agents such as organic stress reducers including trehalose modulate the effect of the cyclooxygenase-2 (COX-2) catalytic pathway [20]. In experimental murine models, the polysaccharide reduced concentrations of pro-inflammatory markers leading to a reversal of SAH induced vasospasm. Of the multiple markers that trehalose modulates is endothelin-1, which modulates vasoconstriction [91, 92], via its effects on nitric oxide, fibrosis, tissue growth, and inflammation [92]. Consequently, it has been the target of major studies on the treatment of vasospasm [114]. The reduction in plasma arachidonic acid is also thought to mediate these experimental findings. Molecular hydrogen has been found to be useful experimentally to decrease vasospasm and peroxidation [115]. These evidences along with other recent experimental findings suggest a multifactorial process that precedes CVS and clinical neurological dysfunction, which may or may not be directly related. 

Myeloperoxidase (MPO), a marker of inflammation in coronary artery disease, is thought to be implicated in the inflammatory process of CVS [116]. MPO is a lysosomal enzyme that is a component of the respiratory burst in leukocyte antibacterial function. The enzyme catalyzes the production of hypohalous acids like hypoclorous acid (HOCl), which modify cellular membranes [117]. In reports by Lim and colleagues, the biomarker demonstrated elevations in CSF concentrations that mirror the onset of CBF abnormalities in CVS, supporting MPO as a surrogate predictor of CVS after SAH. Because of to its role in inflammation, its upregulation is most likely related to antecedent leukocyte recruitment [116].

### 3.4. DCI Has an Evolving Pathophysiology

The mortality that ensues after CVS is often due to delayed cerebral ischemia (DCI). DCI is defined as new focal neurological deficits (symptomatic vasospasm) or ischemic focus in the setting of radiographic vasospasm [118]. While the relationship between inflammation and CVS is irrefutable, it is unlikely to be the single perpetrator behind delayed cerebral ischemia (DCI) and subsequent neurologic decline. Results from the CONSCIOUS trials assessed the effects of the endothelin-1 receptor antagonist Clazosentan after SAH, and while there was significant improvement in angiographic CVS, neurological dysfunction was only minimally affected [119]. Hansen-Schwartz and colleagues concluded that these results point to a more extensive implication of inflammation in the pathophysiology of post-SAH neurological dysfunction. Hansen-Schwartz also argued that the lack of temporal alignment between angiographic CVS and DCI symptoms suggest the need for further studies [120].

Cortical spreading depolarization (CSD), intravascular microthrombosis, and early brain injury have all received traction in the field as alternate theories of CVS [120–124]. In the immediate posthemorrhage period, rapid increases in cerebral edema [125], ICP [126, 127], and changes in cerebral autoregulation [128] are all thought to disrupt the homeostatic milieu resulting in early brain injury. This injury fosters an environment for cerebral ischemia with altered perfusion and hypoxia. Transient and sporadic neuroelectrical disruptions also occur in the injured brain after acute injury [129]. Through experimental murine models, researchers have demonstrated that these brief episodes of electrical dampening differentially affect cerebral perfusion resulting in either hyperperfusion or hypoperfusion. This hemodynamic imbalance propagates damage created during early brain injury [130]. Finally, correlations between elevated procoagulant factors and DCI occurrence have strongly suggested a role for microthrombosis in the pathophysiology [131]. The detection of microthrombi in postmortem analysis [132] and a 70% prevalence of microthrombi in SAH patients [133] also point to the involvement of the coagulation cascade in the development of DCI. It is now more likely that CVS is one of many factors that play some part in the neurological decline consistent with DCI. 

### 3.5. New Research

Current clinical trials are investigating possible contributing factors antecedent to DCI and DIND. Most notably, the EARLYDRAIN and LUMAS trials evaluate early evacuation of CSF through a lumbar drain [134, 135]. The LUMAS trial evaluated 210 patients with aSAH and randomized 105 to receive standard therapy and a lumbar drain, with the remaining 105 randomized to the control group receiving only standard management. The authors found that lumbar drainage of CSF after aSAH was associated with reduced DIND and improved early clinical outcome but had no difference in outcomes at 6 months. Preliminary studies have also shown efficacy using a kinetic treatment, intrathecal thrombolytic therapy in conjunction with head shaking. In this small study, 9 patients were selected to receive multimodal treatment while 11 received standard therapy. After 14 days of observation, there was decreased DIND and vasospasm related infarct in the intrathecal thrombolytic therapy group compared to the control [136]. Lastly, the depolarizations in ischaemia after aneurysmal subarachnoid haemorrhage (DISCHARGE-1) trial are an ongoing multicenter diagnostic phase III study that assesses electrical depression of the cerebral cortex after aSAH. The aim is to calculate the sensitivity and specificity of the duration of cortical spreading depression as a proxy for DCI after aSAH. As we look to future research, it is hoped that these theories may become incorporated in to standard therapies for the treatment of CVS. 

## 4. Conclusion

From all indications, one of the major limiting factors in the management of CVS is an understanding of the extent of early brain injury secondary to SAH. Various studies have proven the relationship between vasospasm and accompanying neurological decline. We are currently limited by the difficulty in consistently and accurately diagnosing and treating affected patients. It is unlikely that there will be a “silver bullet” treatment for CVS and DCI. The role of inflammation in CVS represents a possible mechanism behind the development of DCI after SAH, and targeting the inflammatory process has potential to significantly reduce CVS, though further studies are necessary. Cerebral vasospasm continues to be regarded as an important factor in the progression to DCI in patients following SAH. It cannot be however regarded as the single factor behind the neurological decline in this patient cohort. 

## Figures and Tables

**Table 1:** Landmark discoveries of the relationship between vasospasm and inflammation in the last halfcentury.

History of the relationship between inflammatory markers and cerebral vasospasm: a look at contributions to the literature			
Author (year)	Inflammatory parameter assessed	Model	Findings
Walton (1955) [52]	Fever	Human	SAH patients with fever have decreased survival
Rousseaux et al. (1980) [53]	Fever	Human	SAH patients with fever have decreased survival
Spallone et al. (1987) [54]	Leukocytosis	Human	SAH is accompanied by leukocytosis
Mathiesen et al. (1990) [137]	Neopterin	Human	CSF neopterin levels rise with SAH
Minami et al. (1991) [103]	LTC4	Canine	LTC4 expression increases with SAH
Peterson et al. (1990) [138]	Cell free blood components	Canine	Subarachnoid foreign bodies induce angiographic BA vasospasm
Edwards et al. (1992) [57]	EDRF	Porcine	Adventitial hemoglobin reduces EDRF concentrations
Onda et al. (1999) [139]	MCP-1, cystatin B, inter-alpha-trypsin inhibitor, serum amyloid A protein, and GP130	Canine	SAH increases expression of inflammatory markers genes
Fabender et al., (2000) [9]	ET1, IL-6, and TNF*α*	In vitro	Leukocytes activated by incubation with blood release inflammatory markers
Aihara et al. (2001) [140]	Il-1A, Il-6, Il-6, Il10, VCAM-1, TGF-ss, and bFGF	Canine	SAH increases expression of markers with BA vasospasm
McGirt et al. (2002) [141]	MM-9, VEGF, and vWF	Human	Increases in VEGF, MMP-9, and VEGF precede angiographic vasospasm
Sasaki et al. (2004) [142]	IL-1a, IL-1b, and IL-8	Canine	Hemolysate increased inflammatory marker expression by MAPK mediated pathways
Recinos et al. (2006) [19]	LPS	Leporine	LPS induces BA narrowing and clinical CVS
Zhou et al. (2007) [143]	TNF*α*, ICAM-1, and MPO	Leporine	Hemolysate increases NF-Kb DNA binding activity with increase in inflammatory markers and angiographic BA vasospasm
Jȩdrzejowska-Szypułka et al. (2009) [68]	IL-1B	Murine	IL-1B activity increased with hemolysate and reversed with IL-1B antibodies
Wang et al. (2010) [144]	CD34	Murine	CD34 expression increases with SAH at the peak of vasospasm
Wirrig et al. (2011) [145]	SPC	Murine	SPC increases MCP-1 and acts as inflammatory mediator in CVS

LTC4: leukotriene C4, MCP-1: monocyte chemotactic protein 1, ET1: endothelin 1, TNF*α*: tumor necrosis factor alpha, MMP9: matrix metalloproteinase, bFGF: basic fibroblast growth factor, EDRF: endothelium derived relaxing factor, IL1: interleukin 1, LPS: lipopolysaccharide, ICAM-1: intercellular adhesion molecule 1, MPO: myeloperoxidase, vWF: von Willebrand's factor, CD34: cluster of differentiation 34, SPC: sphyngosylphosphorylcholine, BA: basilar artery, and GP130: glycoprotein 130.

**Table 2:** Early studies report improvement in experimental proxies of CVS in various models.

Studies involving modifications of the inflammatory process with effects on CVS			
Author (year)	Parameter assessed	Model	Findings
Tokiyoshi et al. (1991) [146]	Symptomatic vasospasm	Human	TXA2 synthetase inhibition decreased symptomatic vasospasm
Lin et al. (2005) [67]	ACA diameter	Murine	Anti-E selectin mAb prevents SAH induced angiographic vasospasm
Fei and Golwa (2007) [101]	MCA velocity	Human	Topical dexamethasone prevents angiographic vasospasm
Iseda et al. (2007) [147]	BA diameter, IL-1B	Leporine	Caspase inhibitor (Z-VAD-FMK) decreased angiographic BA vasospasm and IL-1B levels
Lin et al. (2007) [66]	ICAM-1, BA diameter	Leporine	Endothelin converting enzyme inhibitor (CGS 26303) decreases ICAM-1 levels and BA vasospasm
Chen et al. (2008) [69]	JAK2	Leporine	JAK2 inhibitor (AG490) decreases JAK2 activation and angiographic BA vasospasm
Yoshimoto et al. (2009) [148]	Angiography	Human	Cilostazol prevents angiographic vasospasm after SAH
Wu et al. (2010) [149]	TLR4, TNF1	In vitro	PPAR gamma agonist decreases TLR4 expression and cytokine release
Chang et al. (2010) [150]	ICAM-1, VCAM-1, E-Selectin, and BA diameter	Murine	6-MP decreases ICAM-1 and E-Selectin and increases angiographic BA vasospasm after SAH

TXA2: thromboxane A2, PAF: platelet activating factor, LFA-1: lymphocyte function associated antigen, NO: nitrous oxide, JNK: c-Jun N terminal kinase, mAb: monoclonal antibody, HMG-CoA: 3-hydroxy-3-methylglutaryl-coenzyme A, JAK2: Janus kinase 2, PDE: phosphodiesterase, 6-MP: 6-mercaptopurine, and PPAR gamma: peroxisome proliferator activated receptor gamma.

**Table 3:** Recent studies have shown improvements in clinical and experimental vasospasm with the use of other various pharmacological additives.

Recent assessments of potential treatments for vasospasm after SAH				
Author (year)	Intervention	Parameter altered/assessed	Model	Findings
Meyers and Connolly (2011) [119]	Endothelial receptor antagonist	Delayed ischemic neurological decline	Human	Endothelial receptor antagonists (Clazosentan) have no effect on vasospasm related morbidity and mortality
Fathi et al. (2011) [89]	Sodium nitrate	Arterial diameter	Primates	IV sodium nitrite reverses CVS after SAH
Muehlschlegel et al. (2011) [151]	Dantrolene	TCD	Human	IV dantrolene decreases CBF after SAH
Echigo et al. (2012) [20]	Trehalose	NF-Kb, ET-1, COX-2, and NO_*x*_	Leporine	Trehalose decreases lipid peroxidation, arachidonic acid release, vasospasm, and inflammatory markers after SAH
Hong et al. (2012) [115]	Hydrogen rich saline	SOD, GPx, and malondialdehyde	Murine	Molecular hydrogen reduces peroxidation and vasospasm after SAH
Pradilla et al. (2012) [23]	L-Citrulline	BA diameter, neurobehaviour, and NOS expression	Murine	Systemic L-citrulline prevents angiographic BA vasospasm and improves neurobehaviour and NOS expression after SAH
Zhang et al. (2012) [152]	mTOR inhibition	BA diameter, appetite, and activity scores	Canine	mTOR inhibitor (rapamycin) reduced CVS after SAH

SOD: superoxide dismutase, CBF: cerebral blood flow, GPx: glutathione peroxidase, NOS: nitric oxide synthase, BA: basilar artery, mTOR: mammalian target of rapamycin, and TCD: transcranial Doppler.

## Abbreviations

CVS: Cerebral vasospasm
DCI: Delayed cerebral ischemia
DIND: Delayed ischemic neurological decline
aSAH: Aneurysmal subarachnoid hemorrhage
TBI: Traumatic brain injury
CTA: Computed tomography angiography
PET: Positron emission tomography
SPECT: Single photon emission computed tomography
LTC4: Leukotriene C4
MCP1: Monocyte chemotactic protein 1
ET1: Endothelin 1
TNF*α*: Tumor necrosis factor alpha
MMP9: Matrix metalloproteinase
bFGF: Basic fibroblast growth factor
EDRF: Endothelium derived relaxing factor
IL1: Interleukin 1
LPS: Lipopolysaccharide
ICAM-1: Intercellular adhesion molecule 1
MPO: Myeloperoxidase
vWF: Von Willebrand's factor
CD34: Cluster of differentiation 34
SPC: Sphyngosylphosphorylcholine
BA: Basilar artery
GP130: Glycoprotein 130
TXA2: Thromboxane A2
PAF: Platelet activating factor
LFA-1: Lymphocyte function associated antigen
NO: Hitrous oxide
JNK: c-Jun N terminal kinase
mAb: Honoclonal antibody
HMG-CoA: 3-Hydroxy-3-methylglutaryl-coenzyme A
JAK2: Janus kinase 2
PDE: Phosphodiesterase
6-MP: 6-Mercaptopurine,
PPAR gamma: Peroxisome proliferator activated receptor gamma
SOD: Superoxide dismutase
CBF: Cerebral blood flow
GPx: Glutathione peroxidase
NOS: Nitric oxide synthase
BA: Basilar artery
mTOR: Mammalian target of rapamycin.

## References

[1] Velat GJ, Kimball MM, Mocco J, Hoh BL (2011). Vasospasm after aneurysmal subarachnoid hemorrhage: review of randomized controlled trials and meta-analyses in the literature. *World Neurosurgery*.

[2] Mayberg MR, Batjer HH, Dacey R (1994). Guidelines for the management of aneurysmal subarachnoid hemorrhage: a statement for healthcare professionals from a special writing group of the Stroke Council, American Heart Association. *Circulation*.

[3] Kassell NF, Sasaki T, Colohan ART, Nazar G (1985). Cerebral vasospasm following aneurysmal subarachnoid hemorrhage. *Stroke*.

[4] Weir B, Macdonald RL, Stoodley M (1999). Etiology of cerebral vasospasm. *Acta Neurochirurgica, Supplement*.

[5] Haley EC, Kassell NF, Torner JC (1992). The international cooperative study on the timing of aneurysm surgery: the North American experience. *Stroke*.

[6] Mayberg MR, Batjer HH, Dacey R (1994). Guidelines for the management of aneurysmal subarachnoid hemorrhage: a statement for healthcare professionals from a special writing group of the Stroke Council, American Heart Association. *Stroke*.

[7] Bavbek M, Polin R, Kwan A-L, Arthur AS, Kassell NF, Lee KS (1998). Monoclonal antibodies against ICAM-1 and CD18 attenuate cerebral vasospasm after experimental subarachnoid hemorrhage in rabbits. *Stroke*.

[8] Thai Q-A, Oshiro EM, Tamargo RJ (1999). Inhibition of experimental vasospasm in rats with the periadventitial administration of ibuprofen using controlled-release polymers. *Stroke*.

[9] Fabender K, Hodapp B, Rossol S (2000). Endothelin-1 in subarachnoid hemorrhage: an acute-phase reactant produced by cerebrospinal fluid leukocytes. *Stroke*.

[10] Clatterbuck RE, Gailloud P, Ogata L (2003). Prevention of cerebral vasospasm by a humanized anti-CD11/CD18 monoclonal antibody administered after experimental subarachnoid hemorrhage in nonhuman primates. *Journal of Neurosurgery*.

[11] Dumont AS, Dumont RJ, Chow MM (2003). Cerebral vasospasm after subarachnoid hemorrhage: putative role of inflammation. *Neurosurgery*.

[12] McGirt MJ, Mavropoulos JC, McGirt LY (2003). Leukocytosis as an independent risk factor for cerebral vasospasm following aneurysmal subarachnoid hemorrhage. *Journal of Neurosurgery*.

[13] Ulbrich H, Eriksson EE, Lindbom L (2003). Leukocyte and endothelial cell adhesion molecules as targets for therapeutic interventions in inflammatory disease. *Trends in Pharmacological Sciences*.

[14] Frazier JL, Pradilla G, Wang PP, Tamargo RJ (2004). Inhibition of cerebral vasospasm by intracranial delivery of ibuprofen from a controlled-release polymer in a rabbit model of subarachnoid hemorrhage. *Journal of Neurosurgery*.

[15] Pradilla G, Wang PP, Legnani FG, Ogata L, Dietsch GN, Tamargo RJ (2004). Prevention of vasospasm by anti-CD11/CD18 monoclonal antibody therapy following subarachnoid hemorrhage in rabbits. *Journal of Neurosurgery*.

[16] Keiper T, Santoso S, Nawroth PP, Orlova V, Chavakis T (2005). The role of junctional adhesion molecules in cell-cell interactions. *Histology and Histopathology*.

[17] Pradilla G, Thai Q-A, Legnani FG (2005). Local delivery of ibuprofen via controlled-release polymers prevents angiographic vasospasm in a monkey model of subarachnoid hemorrhage. *Neurosurgery*.

[18] Gallia GL, Tamargo RJ (2006). Leukocyte-endothelial cell interactions in chronic vasospasm after subarachnoid hemorrhage. *Neurological Research*.

[19] Recinos PF, Pradilla G, Thai Q-A, Perez M, Hdeib AM, Tamargo RJ (2006). Controlled release of lipopolysaccharide in the subarachnoid space of rabbits induces chronic vasospasm in the absence of blood. *Surgical Neurology*.

[20] Echigo R, Shimohata N, Karatsu K, Yano F, Kayasuga-Kariya Y, Fujisawa A (2012). Trehalose treatment suppresses inflammation, oxidative stress, and vasospasm induced by experimental subarachnoid hemorrhage. *Journal of Translational Medicine*.

[21] Takeuchi H, Tanabe M, Okamoto H, Yamazaki M (1999). Effects of thromboxane synthetase inhibitor (RS-5186) on experimentally-induced cerebral vasospasm. *Neurological Research*.

[22] Jadhav VD, Jabre A, Lee TJ-F (2008). Effect of phospholipase C blockade on cerebral vasospasm. *Cerebrovascular Diseases*.

[23] Pradilla G, Garzon-Muvdi T, Ruzevick JJ (2012). Systemic L-citrulline prevents cerebral vasospasm in haptoglobin 2-2 transgenic mice after subarachnoid hemorrhage. *Neurosurgery*.

[24] Vellimana AK, Milner E, Azad TD (2011). Endothelial nitric oxide synthase mediates endogenous protection against subarachnoid hemorrhage-induced cerebral vasospasm. *Stroke*.

[25] Pluta RM (2005). Delayed cerebral vasospasm and nitric oxide: review, new hypothesis, and proposed treatment. *Pharmacology and Therapeutics*.

[26] Suhardja A (2004). Mechanisms of disease: roles of nitric oxide and endothelin-1 in delayed cerebral vasospasm produced by aneurysmal subarachnoid hemorrhage. *Nature Clinical Practice Cardiovascular Medicine*.

[27] Momin EN, Schwab KE, Chaichana KL, Miller-Lotan R, Levy AP, Tamargo RJ (2009). Controlled delivery of nitric oxide inhibits leukocyte migration and prevents vasospasm in haptoglobin 2-2 mice after subarachnoid hemorrhage. *Neurosurgery*.

[28] Capampangan DJ, Wellik KE, Aguilar MI, Demaerschalk BM, Wingerchuk DM (2008). Does prophylactic postoperative hypervolemic therapy prevent cerebral vasospasm and improve clinical outcome after aneurysmal subarachnoid hemorrhage?. *Neurologist*.

[29] Vorkapic P, Bevan JA, Bevan RD (1991). Longitudinal in vivo and in vitro time-course study of chronic cerebrovasospasm in the rabbit basilar artery. *Neurosurgical Review*.

[30] Weir B, Grace M, Hansen J, Rothberg C (1978). Time course of vasospasm in man. *Journal of Neurosurgery*.

[31] Mills JN, Mehta V, Russin J, Amar AP, Rajamohan A, Mack WJ (2013). Advanced imaging modalities in the detection of cerebral vasospasm. *Neurology Research International*.

[32] Ecker A, Riemenschneider PA (1951). Arteriographic demonstration of spasm of the intracranial arteries, with special reference to saccular arterial aneurysms. *Journal of Neurosurgery*.

[33] Perez-Arjona EA, Delproposto Z, Sehgal V, Fessler RD (2002). New techniques in cerebral imaging. *Neurological Research*.

[34] Janjua N, Mayer SA (2003). Cerebral vasospasm after subarachnoid hemorrhage. *Current Opinion in Critical Care*.

[35] Greenberg ED, Gold R, Reichman M (2010). Diagnostic accuracy of CT angiography and CT perfusion for cerebral vasospasm: a meta-analysis. *American Journal of Neuroradiology*.

[36] Dehdashti AR, Rufenacht DA, Delavelle J, Reverdin A, De Tribolet N (2003). Therapeutic decision and management of aneurysmal subarachnoid haemorrhage based on computed tomographic angiography. *British Journal of Neurosurgery*.

[37] Anderson GB, Findlay JM, Steinke DE, Ashforth R (1997). Experience with computed tomographic angiography for the detection of intracranial aneurysms in the setting of acute subarachnoid hemorrhage. *Neurosurgery*.

[38] Chen F, Wang X, Wu B (2011). Neuroimaging research on cerebrovascular spasm and its current progress. *Acta Neurochirurgica. Supplement*.

[39] Tamatani S, Sasaki O, Takeuchi S, Fujii Y, Koike T, Tanaka R (1997). Detection of delayed cerebral vasospasm, after rupture of intracranial aneurysms, by magnetic resonance angiography. *Neurosurgery*.

[40] Grandin CB, Cosnard G, Hammer F, Duprez TP, Stroobandt G, Mathurin P (2000). Vasospasm after subarachnoid hemorrhage: diagnosis with MR angiography. *American Journal of Neuroradiology*.

[41] Dalessandri K, St. John JN, Carson SN (1981). Correction and monitoring of postoperative cerebral vasospasm. *Angiology*.

[42] Aaslid R, Markwalder TM, Nornes H (1982). Noninvasive transcranial Doppler ultrasound recording of flow velocity in basal cerebral arteries. *Journal of Neurosurgery*.

[43] Suarez JI, Qureshi AI, Yahia AB (2002). Symptomatic vasospasm diagnosis after subarachnoid hemorrhage: evaluation of transcranial Doppler ultrasound and cerebral angiography as related to compromised vascular distribution. *Critical Care Medicine*.

[44] Burch CM, Wozniak MA, Sloan MA (1996). Detection of intracranial internal carotid artery and middle cerebral artery vasospasm following subarachnoid hemorrhage. *Journal of Neuroimaging*.

[45] Wozniak MA, Sloan MA, Rothman MI (1996). Detection of vasospasm by transcranial Doppler sonography: the challenges of the anterior and posterior cerebral arteries. *Journal of Neuroimaging*.

[46] Lindegaard KF, Nornes H, Bakke SJ, Sorteberg W, Nakstad P (1988). Cerebral vasospasm after subarachnoid haemorrhage investigated by means of transcranial Doppler ultrasound. *Acta Neurochirurgica, Supplement*.

[47] Okada Y, Shima T, Nishida M (1999). Comparison of transcranial doppler investigation of aneurysmal vasospasm with digital subtraction angiographic and clinical findings. *Neurosurgery*.

[48] White H, Venkatesh B (2006). Applications of transcranial Doppler in the ICU: a review. *Intensive Care Medicine*.

[49] Lysakowski C, Walder B, Costanza MC, Tramèr MR (2001). Transcranial Doppler versus angiography in patients with vasospasm due to a ruptured cerebral aneurysm: a systematic review. *Stroke*.

[50] Sloan MA, Haley EC, Kassel NF (1989). Sensitivity and specificity of transcranial Doppler ultrasonography in the diagnosis of vasospasm following subarachnoid hemorrhage. *Neurology*.

[51] Liu-DeRyke X, Rhoney DH (2006). Cerebral vasospasm after aneurysmal subarachnoid hemorrhage: an overview of pharmacologic management. *Pharmacotherapy*.

[52] Walton JN (1955). The prognosis and management of subarachnoid haemorrhage. *Canadian Medical Association Journal*.

[53] Rousseaux P, Scherpereel B, Bernard MH, Graftieaux JP, Guyot JF (1980). Fever and cerebral vasospasm in ruptured intracranial aneurysms. *Surgical Neurology*.

[54] Spallone A, Acqui M, Pastore FS, Guidetti B (1987). Relationship between leukocytosis and ischemic complications following aneurysmal subarachnoid hemorrhage. *Surgical Neurology*.

[55] Pradilla G, Chaichana KL, Hoang S, Huang J, Tamargo RJ (2010). Inflammation and cerebral vasospasm after subarachnoid hemorrhage. *Neurosurgery Clinics of North America*.

[56] Chaichana KL, Pradilla G, Huang J, Tamargo RJ (2010). Role of inflammation (leukocyte-endothelial cell interactions) in vasospasm after subarachnoid hemorrhage. *World Neurosurgery*.

[57] Edwards DH, Byrne JV, Griffith TM (1992). The effect of chronic subarachnoid hemorrhage on basal endothelium-derived relaxing factor activity in intrathecal cerebral arteries. *Journal of Neurosurgery*.

[58] Agil A, Fuller CJ, Jialal I (1995). Susceptibility of plasma to ferrous iron/hydrogen peroxide-mediated oxidation: demonstration of a possible Fenton reaction. *Clinical Chemistry*.

[59] Griffith TM, Edwards DH, Lewis MJ, Newby AC, Henderson AH (1984). The nature of endothelium-derived vascular relaxant factor. *Nature*.

[60] Gutteridge JMC (1995). Lipid peroxidation and antioxidants as biomarkers of tissue damage. *Clinical Chemistry*.

[61] Kolias AG, Sen J, Belli A (2009). Pathogenesis of cerebral vasospasm following aneurysmal subarachnoid hemorrhage: putative mechanisms and novel approaches. *Journal of Neuroscience Research*.

[62] Ayer RE, Zhang JH (2008). Oxidative stress in subarachnoid haemorrhage: significance in acute brain injury and vasospasm. *Acta Neurochirurgica, Supplementum*.

[63] Maeda Y, Hirano K, Nishimura J, Sasaki T, Kanaide H (2004). Endothelial dysfunction and altered bradykinin response due to oxidative stress induced by serum deprivation in the bovine cerebral artery. *European Journal of Pharmacology*.

[64] Provencio JJ, Vora N (2005). Subarachnoid hemorrhage and inflammation: bench to bedside and back. *Seminars in Neurology*.

[65] Chaichana KL, Levy AP, Miller-Lotan R, Shakur S, Tamargo RJ (2007). Haptoglobin 2-2 genotype determines chronic vasospasm after experimental subarachnoid hemorrhage. *Stroke*.

[66] Lin C-L, Kwan A-L, Dumont AS (2007). Attenuation of experimental subarachnoid hemorrhage-induced increases in circulating intercellular adhesion molecule-1 and cerebral vasospasm by the endothelin-converting enzyme inhibitor CGS 26303. *Journal of Neurosurgery*.

[67] Lin C-L, Dumont AS, Calisaneller T, Kwan A-L, Hwong S-L, Lee KS (2005). Monoclonal antibody against E selectin attenuates subarachnoid hemorrhage-induced cerebral vasospasm. *Surgical Neurology*.

[68] Jȩdrzejowska-Szypułka H, Larysz-Brysz M, Kukla M, Śnietura M, Lewin-Kowalik J (2009). Neutralization of interleukin-1*β* reduces vasospasm and alters cerebral blood vessel density following experimental subarachnoid hemorrhage in rats. *Current Neurovascular Research*.

[69] Chen G, Wu J, Sun C (2008). Potential role of JAK2 in cerebral vasospasm after experimental subarachnoid hemorrhage. *Brain Research*.

[70] Bowman G, Bonneau RH, Chinchilli VM, Tracey KJ, Cockroft KM (2006). A novel inhibitor of inflammatory cytokine production (CNI-1493) reduces rodent post-hemorrhagic vasospasm. *Neurocritical Care*.

[71] Yatsushige H, Yamaguchi M, Zhou C, Calvert JW, Zhang JH (2005). Role of c-Jun N-terminal kinase in cerebral vasospasm after experimental subarachnoid hemorrhage. *Stroke*.

[72] Muller WA (2011). Mechanisms of leukocyte transendothelial migration. *Annual Review of Pathology*.

[73] Iigo Y, Suematsu M, Higashida T (1997). Constitutive expression of ICAM-1 in rat microvascular systems analyzed by laser confocal microscopy. *American Journal of Physiology*.

[74] Klein CL, Bittinger F, Kohler H (1995). Comparative studies on vascular endothelium in vitro: 3. Effects of cytokines on the expression of E-Selectin, ICAM-1 and VCAM-1 by cultured human endothelial cells obtained from different passages. *Pathobiology*.

[75] Scholz D, Devaux B, Hirche A (1996). Expression of adhesion molecules is specific and time-dependent in cytokine-stimulated endothelial cells in culture. *Cell and Tissue Research*.

[76] Hirashima Y, Nakamura S, Endo S, Kuwayama N, Naruse Y, Takaku A (1997). Elevation of platelet activating factor, inflammatory cytokines, and coagulation factors in the internal jugular vein of patients with subarachnoid hemorrhage. *Neurochemical Research*.

[77] Fassbender K, Hodapp B, Rossol S (2001). Inflammatory cytokines in subarachnoid haemorrhage: association with abnormal blood flow velocities in basal cerebral arteries. *Journal of Neurology Neurosurgery and Psychiatry*.

[78] Muroi C, Burkhardt JK, Hugelshofer M, Seule M, Mishima K, Keller E (2012). Magnesium and the inflammatory response: potential pathophysiological implications in the management of patients with aneurysmal subarachnoid hemorrhage?. *Magnesium Research*.

[79] Ni W, Gu YX, Song DL, Leng B, Li PL, Mao Y (2011). The relationship between IL-6 in CSF and occurrence of vasospasm after subarachnoid hemorrhage. *Acta Neurochirurgica. Supplement*.

[80] Hendryk S, Jarzab B, Josko J (2004). Increase of the IL-1*β* and IL-6 levels in CSF in patients with vasospasm following aneurysmal SAH. *Neuroendocrinology Letters*.

[81] Mathieson T, Edner G, Ulfarsson E, Andersson B (1997). Cerebrospinal fluid interleukin-1 receptor antagonist and tumor necrosis factor-*α* following subarachnoid hemorrhage. *Journal of Neurosurgery*.

[82] Sarrafzadeh A, Schlenk F, Gericke C, Vajkoczy P (2010). Relevance of cerebral interleukin-6 after aneurysmal subarachnoid hemorrhage. *Neurocritical Care*.

[83] Rothoerl RD, Axmann C, Pina A-L, Woertgen C, Brawanski A (2006). Possible role of the C-reactive protein and white blood cell count in the pathogenesis of cerebral vasospasm following aneurysmal subarachnoid hemorrhage. *Journal of Neurosurgical Anesthesiology*.

[84] Parkinson D, Stephensen S (1984). Leukocytosis and subarachnoid hemorrhage. *Surgical Neurology*.

[85] Crompton MR (1964). The pathogenesis of cerebral infarction following the rupture of cerebral berry aneurysms. *Brain*.

[86] Hughes JT, Schianchi PM (1978). Cerebral artery spasm. A histological study at necropsy of the blood vessels in cases of subarachnoid hemorrhage. *Journal of Neurosurgery*.

[87] Fraticelli AT, Cholley BP, Losser M-R, Maurice J-PS, Payen D (2008). Milrinone for the treatment of cerebral vasospasm after aneurysmal subarachnoid hemorrhage. *Stroke*.

[88] Biondi A, Ricciardi GK, Puybasset L (2004). Intra-arterial nimodipine for the treatment of symptomatic cerebral vasospasm after aneurysmal subarachnoid hemorrhage: preliminary results. *American Journal of Neuroradiology*.

[89] Fathi AR, Pluta RM, Bakhtian KD, Qi M, Lonser RR (2011). Reversal of cerebral vasospasm via intravenous sodium nitrite after subarachnoid hemorrhage in primates: laboratory investigation. *Journal of Neurosurgery*.

[90] Thampatty BP, Sherwood PR, Gallek MJ (2011). Role of Endothelin-1 in human aneurysmal subarachnoid hemorrhage: associations with vasospasm and delayed cerebral ischemia. *Neurocritical Care*.

[91] Asano T, Ikegaki I, Suzuki Y, Satoh S, Shibuya M (1989). Endothelin and the production of cerebral vasospasm in dogs. *Biochemical and Biophysical Research Communications*.

[92] Zimmermann M, Seifert V (2004). Endothelin receptor antagonists and cerebral vasospasm. *Clinical Autonomic Research*.

[93] Thorin E, Nguyen T-D, Bouthillier A (1998). Control of vascular tone by endogenous endothelin-1 in human pial arteries. *Stroke*.

[94] Bian K, Murad F (2003). Nitric oxide (NO)—biogeneration, regulation, and relevence to human diseases. *Frontiers in Bioscience*.

[95] Mascia L, Fedorko L, Stewart DJ (2001). Temporal relationship between endothelin-1 concentrations and cerebral vasospasm in patients with aneurysmal subarachnoid hemorrhage. *Stroke*.

[96] Kwan A-L, Solenski NJ, Kassell NF, Lee KS (1997). Inhibition of nitric oxide generation and lipid peroxidation attenuates hemolysate-induced injury to cerebrovascular endothelium. *Acta Neurochirurgica*.

[97] Pluta RM, Thompson BG, Dawson TM, Snyder SH, Boock RJ, Oldfield EH (1996). Loss of nitric oxide synthase immunoreactivity in cerebral vasospasm. *Journal of Neurosurgery*.

[98] Iuliano BA, Pluta RM, Jung C, Oldfield EH (2004). Endothelial dysfunction in a primate model of cerebral vasospasm. *Journal of Neurosurgery*.

[99] Pluta RM, Afshar JKB, Boock RJ, Oldfield EH (1998). Temporal changes in perivascular concentrations of oxyhemoglobin, deoxyhemoglobin, and methemoglobin after subarachnoid hemorrhage. *Journal of Neurosurgery*.

[100] Peterson JW, Nishizawa S, Hackett JD, Bun T, Teramura A, Zervas NT (1990). Cyclosporine A reduces cerebral vasospasm after subarachnoid hemorrhage in dogs. *Stroke*.

[101] Fei L, Golwa F (2007). Topical application of dexamethasone to prevent cerebral vasospasm after aneurysmal subarachnoid haemorrhage: a pilot study. *Clinical Drug Investigation*.

[102] Nagata K, Sasaki T, Iwama J (1993). Failure of FK-506, a new immunosuppressant, to prevent cerebral vasospasm in a canine two-hemorrhage model. *Journal of Neurosurgery*.

[103] Minami N, Tani E, Yokota M, Maeda Y, Yamaura I (1991). Immunohistochemistry of leukotriene C4 in experimental cerebral vasospasm. *Acta Neuropathologica*.

[104] Sugawara T, Ayer R, Jadhav V, Chen W, Tsubokawa T, Zhang JH (2011). Mechanisms of statin treatment in cerebral vasospasm. *Acta neurochirurgica. Supplement*.

[105] Lynch JR, Wang H, McGirt MJ (2005). Simvastatin reduces vasospasm after aneurysmal subarachnoid hemorrhage: results of a pilot randomized clinical trial. *Stroke*.

[106] Trimble JL, Kockler DR (2007). Statin treatment of cerebral vasospasm after aneurysmal subarachnoid hemorrhage. *Annals of Pharmacotherapy*.

[107] Bowman G, Dixit S, Bonneau RH (2004). Neutralizing antibody against interleukin-6 attenuates posthemorrhagic vasospasm in the rat femoral artery model. *Neurosurgery*.

[108] Yahia AM, Kirmani JF, Qureshi AI, Guterman LR, Hopkins LN (2005). The safety and feasibility of continuous intravenous magnesium sulfate for prevention of cerebral vasospasm in aneurysmal subarachnoid hemorrhage. *Neurocritical Care*.

[109] Shah QA, Memon MZ, Suri MFK (2009). Super-selective intra-arterial magnesium sulfate in combination with nicardipine for the treatment of cerebral vasospasm in patients with subarachnoid hemorrhage. *Neurocritical Care*.

[110] Pyne GJ, Cadoux-Hudson TAD, Clark JF (2001). Magnesium protection against in vitro cerebral vasospasm after subarachnoid haemorrhage. *British Journal of Neurosurgery*.

[111] Mori K, Yamamoto T, Miyazaki M (2012). Effect of intrathecal magnesium sulfate solution injection via a microcatheter in the cisterna magna on cerebral vasospasm in the canine subarachnoid haemorrhage model. *British Journal of Neurosurgery*.

[112] Jeon JS, Sheen SH, Hwang G, Kang SH, Heo DH, Cho YJ (2012). Intravenous magnesium infusion for the prevention of symptomatic cerebral vasospasm after aneurysmal subarachnoid hemorrhage. *Journal of Korean Neurosurgical Society*.

[113] Altura BT, Altura BM (1980). Withdrawal of magnesium causes vasospasm while elevated magnesium produces relaxation of tone in cerebral arteries. *Neuroscience Letters*.

[114] Macdonald RL, Higashida RT, Keller E (2011). Clazosentan, an endothelin receptor antagonist, in patients with aneurysmal subarachnoid haemorrhage undergoing surgical clipping: a randomised, double-blind, placebo-controlled phase 3 trial (CONSCIOUS-2). *The Lancet Neurology*.

[115] Hong Y, Guo S, Chen S, Sun C, Zhang J, Sun X (2012). Beneficial effect of hydrogen-rich saline on cerebral vasospasm after experimental subarachnoid hemorrhage in rats. *Journal of Neuroscience Research*.

[116] Lim M, Bower RS, Wang Y (2012). The predictive value of serum myeloperoxidase for vasospasm in patients with aneurysmal subarachnoid hemorrhage. *Neurosurgical Review*.

[117] Prokopowicz Z, Marcinkiewicz J, Katz DR, Chain BM (2012). Neutrophil myeloperoxidase: soldier and statesman. *Archivum Immunologiae et Therapiae Experimentalis*.

[118] Frontera JA, Fernandez A, Schmidt JM (2009). Defining vasospasm after subarachnoid hemorrhage: what is the most clinically relevant definition?. *Stroke*.

[119] Meyers PM, Connolly ES (2011). Stroke: disappointing results for clazosentan in CONSCIOUS-2. *Nature Reviews Neurology*.

[120] Hansen-Schwartz J, Vajkoczy P, Macdonald RL, Pluta RM, Zhang JH (2007). Cerebral vasospasm: looking beyond vasoconstriction. *Trends in Pharmacological Sciences*.

[121] Leng LZ, Fink ME, Iadecola C (2011). Spreading depolarization: a possible new culprit in the delayed cerebral ischemia of subarachnoid hemorrhage. *Archives of Neurology*.

[122] Muroi C, Seule M, Mishima K, Keller E (2012). Novel treatments for vasospasm after subarachnoid hemorrhage. *Current Opinion in Critical Care*.

[123] Vergouwen MDI, Vermeulen M, Coert BA, Stroes ESG, Roos YBWEM (2008). Microthrombosis after aneurysmal subarachnoid hemorrhage: an additional explanation for delayed cerebral ischemia. *Journal of Cerebral Blood Flow and Metabolism*.

[124] Rowland MJ, Hadjipavlou G, Kelly M, Westbrook J, Pattinson KT (2012). Delayed cerebral ischaemia after subarachnoid haemorrhage: looking beyond vasospasm. *British Journal of Anaesthesia*.

[125] Claassen J, Carhuapoma JR, Kreiter KT, Du EY, Connolly ES, Mayer SA (2002). Global cerebral edema after subarachnoid hemorrhage: frequency, predictors, and impact on outcome. *Stroke*.

[126] Nornes H (1973). The role of intracranial pressure in the arrest of hemorrhage in patients with ruptured intracranial aneurysm. *Journal of Neurosurgery*.

[127] Grote E, Hassler W (1988). The critical first minutes after subarachnoid hemorrhage. *Neurosurgery*.

[128] Rätsep T, Asser T (2001). Cerebral hemodynamic impairment after aneurysmal subarachnoid hemorrhage as evaluated using transcranial Doppler ultrasonography: relationship to delayed cerebral ischemia and clinical outcome. *Journal of Neurosurgery*.

[129] Dreier JP, Woitzik J, Fabricius M (2006). Delayed ischaemic neurological deficits after subarachnoid haemorrhage are associated with clusters of spreading depolarizations. *Brain*.

[130] Dreier JP, Major S, Manning A (2009). Cortical spreading ischaemia is a novel process involved in ischaemic damage in patients with aneurysmal subarachnoid haemorrhage. *Brain*.

[131] Frijns CJM, Fijnheer R, Algra A, Van Mourik JA, Van Gijn J, Rinkel GJE (2006). Early circulating levels of endothelial cell activation markers in aneurysmal subarachnoid haemorrhage: associations with cerebral ischaemic events and outcome. *Journal of Neurology, Neurosurgery and Psychiatry*.

[132] Suzuki S, Kimura M, Souma M, Ohkima H, Iwabuchi T, Shimiz u T (1990). Cerebral microthrombosis in symptomatic cerebral vasospasm—a quantitative histological study in autopsy cases. *Neurologia Medico-Chirurgica*.

[133] Romano JG, Forteza AM, Concha M (2002). Detection of microemboli by transcranial Doppler ultrasonography in aneurysmal subarachnoid hemorrhage. *Neurosurgery*.

[134] Al-Tamimi YZ, Bhargava D, Feltbower RG (2012). Lumbar drainage of cerebrospinal fluid after aneurysmal subarachnoid hemorrhage: a prospective, randomized, controlled trial (LUMAS). *Stroke*.

[135] Bardutzky J, Witsch J, Jüttler E, Schwab S, Vajkoczy P, Wolf S (2011). EARLYDRAIN-outcome after early lumbar CSF-drainage in aneurysmal subarachnoid hemorrhage: study protocol for a randomized controlled trial. *Trials*.

[136] Hänggi D, Eicker S, Beseoglu K, Behr J, Turowski B, Steiger H-J (2009). A multimodal concept in patients after severe aneurysmal subarachnoid hemorrhage: results of a controlled single centre prospective randomized multimodal phase I/II trial on cerebral vasospasm. *Zentralblatt fur Neurochirurgie*.

[137] Mathiesen T, Fuchs D, Wachter H, von Holst H (1990). Increased CSF neopterin levels in subarachnoid hemorrhage. *Journal of Neurosurgery*.

[138] Peterson JW, Kwun B-D, Hackett JD, Zervas NT (1990). The role of inflammation in experimental cerebral vasospasm. *Journal of Neurosurgery*.

[139] Onda H, Kasuya H, Takakura K (1999). Identification of genes differentially expressed in canine vasospastic cerebral arteries after subarachnoid hemorrhage. *Journal of Cerebral Blood Flow and Metabolism*.

[140] Aihara Y, Kasuya H, Onda H, Hori T, Takeda J (2001). Quantitative analysis of gene expressions related to inflammation in canine spastic artery after subarachnoid hemorrhage. *Stroke*.

[141] McGirt MJ, Lynch JR, Blessing R (2002). Serum von Willebrand factor, matrix metalloproteinase-9, and vascular endothelial growth factor levels predict the onset of cerebral vasospasm after aneurysmal subarachnoid hemorrhage. *Neurosurgery*.

[142] Sasaki T, Kasuya H, Onda H (2004). Role of p38 mitogen-activated protein kinase on cerebral vasospasm after subarachnoid hemorrhage. *Stroke*.

[143] Zhou M-L, Shi J-X, Hang C-H (2007). Potential contribution of nuclear factor-*κ*B to cerebral vasospasm after experimental subarachnoid hemorrhage in rabbits. *Journal of Cerebral Blood Flow and Metabolism*.

[144] Wang Z, Wang K-Y, Wu Y, Zhou P, Sun X-O, Chen G (2010). Potential role of CD34 in cerebral vasospasm after experimental subarachnoid hemorrhage in rats. *Cytokine*.

[145] Wirrig C, Hunter I, Mathieson FA, Nixon GF (2011). Sphingosylphosphorylcholine is a proinflammatory mediator in cerebral arteries. *Journal of Cerebral Blood Flow & Metabolism*.

[146] Tokiyoshi K, Ohnishi T, Nii Y (1991). Efficacy and toxicity of thromboxane synthetase inhibitor for cerebral vasospasm after subarachnoid hemorrhage. *Surgical Neurology*.

[147] Iseda K, Ono S, Onoda K (2007). Antivasospastic and antiinflammatory effects of caspase inhibitor in experimental subarachnoid hemorrhage. *Journal of Neurosurgery*.

[148] Yoshimoto T, Shirasaka T, Fujimoto S (2009). Cilostazol may prevent cerebral vasospasm following subarachnoid hemorrhage. *Neurologia Medico-Chirurgica*.

[149] Wu Y, Zhao X-D, Zhuang Z (2010). Peroxisome proliferator-activated receptor gamma agonist rosiglitazone attenuates oxyhemoglobin-induced Toll-like receptor 4 expression in vascular smooth muscle cells. *Brain Research*.

[150] Chang C-Z, Lin C-L, Kassel NF, Kwan A-L, Howng S-L (2010). 6-mercaptopurine attenuates adhesive molecules in experimental vasospasm. *Acta Neurochirurgica*.

[151] Muehlschlegel S, Rordorf G, Sims J (2011). Effects of a single dose of dantrolene in patients with cerebral vasospasm after subarachnoid hemorrhage: a prospective pilot study. *Stroke*.

[152] Zhang W, Khatibi NH, Yamaguchi-Okada M (2012). Mammalian target of rapamycin (mTOR) inhibition reduces cerebral vasospasm following a subarachnoid hemorrhage injury in canines. *Experimental Neurology*.

